# The Accuracy of Electrical Impedance Tomography for Breast Cancer Detection: A Systematic Review and Meta-Analysis

**DOI:** 10.1155/2022/8565490

**Published:** 2022-05-26

**Authors:** Zahra Rezanejad Gatabi, Mehri Mirhoseini, Nasrin Khajeali, Iman Rezanezhad Gatabi, Maedeh Dabbaghianamiri, Sara Dorri

**Affiliations:** ^1^Department of Pharmaceutics, Faculty of Pharmacy, Mazandaran University of Medical Sciences, Sari, Iran; ^2^Amol Faculty of Paramedical Sciences, Mazandaran University of Medical Sciences, Sari, Iran; ^3^Department of Medical Education, Ahvaz Jundishapur, University of Medical Sciences, Ahvaz, Iran; ^4^IRG IP, Dallas, Texas, USA; ^5^Department of Engineering Technology, Texas State University, San Marcos, TX, USA; ^6^Health Information Technology Research Center, Isfahan University of Medical Science, Isfahan, Iran

## Abstract

**Introduction:**

Incidence of breast cancer (BC) in 2020 is about 2.26 million new cases. It is the first common cancer accounting for 11.7% of all cancer worldwide. Disease complications and the mortality rate of breast cancer are highly dependent on the early diagnosis. Therefore, novel human breast-imaging techniques play an important role in minimizing the breast cancer morbidity and mortality rate. Electrical impedance tomography (EIT) is a noninvasive technique to image the breast using the electrical impedance behavior of the body tissues.

**Objectives:**

The aims of this manuscript are as follows: (1) a comprehensive investigation of the accuracy of EIT for breast cancer diagnosis through searching pieces of evidence in the valid databases and (2) meta-analyses of the results.

**Methods:**

The systematic search was performed in the electronic databases including PubMed, Web of Science, EMBASE, Science Direct, ProQuest, Scopus, and Google Scholar without time and language limitation until January 2021. Search terms were “EIT” and “Breast Cancer” with their synonyms. Relevant studies were included based on PRISMA and study objectives. Quality of studies and risk of bias were performed by QUADAS-2 tools. Then, relevant data were extracted in Excel form. The hierarchical/bivariate meta-analysis was performed with “metandi” package for the ROC plot of sensitivity and specificity. Forest plot of the Accuracy index and double arcsine transformations was applied to stabilize the variance. The heterogeneity of the studies was evaluated by the forest plots, *χ*2 test (assuming a significance at the a-level of 10%), and the I^2^ statistic for the Accuracy index.

**Results:**

A total of 4027 articles were found. Finally, 12 of which met our criteria. Overall, these articles included studies of 5487 breast cancer patients. EIT had an overall pooled sensitivity and specificity of 75.88% (95% CI, 61.92% to 85.89%) and 82.04% (95% CI, 69.72% to 90.06%), respectively. The pooled diagnostic odds ratio was 14.37 (95% CI, 6.22% to 33.20%), and the pooled effect of accuracy was 0.79 with 95% CI (0.73, 0.83).

**Conclusions:**

This study showed that EIT can be used as a useful method alongside mammography. EIT sensitivity could not be compared with the sensitivity of MRI, but in terms of specificity, it can be considered as a new method that probably can get more attention. Furthermore, large-scale studies will be needed to support the evidence.

## 1. Introduction

Incidence of breast cancer (BC) in 2020 is about 2.26 million new cases. It is the first common cancer accounting for 11.7% of all cancer worldwide [1]. It is the leading cause of cancer death in most countries [2].

The mortality rate of breast cancer varies from region to region. Compared to the USA and Europe, some Asian countries such as Japan and China have lower mortality rates due to breast cancer. Breast cancer mortality rate is higher than the mortality rate of colorectal cancer (8%) and lower than the mortality rate of lung cancer [3].

Similar to many other forms of cancers, breast cancer is the result of several environmental and hereditary factors. High-fat diets, alcohol intake, tobaccos, hormones, radiation, age, sex, and childbearing are identified as the primary risk factors [4, 5]. Statistical data show that many women with breast tumors refer to the hospital only after they feel a quite large lump. At that stage of the disease, a biopsy is required to determine whether the tumor is cancerous (malignant) or noncancerous (benign). For this purpose, clinicians often perform a fine-needle aspiration or a core biopsy [6]. The type of the tumor determines the subsequent treatments. While the core biopsy is a reliable technique to characterize the tumors, it is not hazard-free. The process requires taking out a considerable amount of tissue from the body at several locations. There are many reports on cancerous tumor adhering to the tip of the biopsy needle and introducing the disease to the upper cutaneous layers [7].

The early diagnosis of breast cancer plays a crucial role in minimizing the mortality rate of the disease. Magnetic resonance imaging (MRI), computed tomography (CT) scan, ultrasound imaging, and mammography are current techniques implemented for diagnostic imaging. Among these techniques, mammography became a standard technique for breast imaging and early-stage detection of carcinomas in the breast [8, 9]. Ultrasound imaging is also employed along with mammography to differentiate suspicious breast lesions [10]. While the mammography technique offers invaluable advantages to decrease mortality [11], it has some drawbacks as well. The disadvantage is that it applies ionizing radiation, which is associated with patient discomfort because of breast tissue compression [12]. In addition to the radiation exposure issues, the mammogram may result in false-positive results. It also has a sensitivity of lower than 100% [13]. Also, MRI has significant disadvantages as well, including its high cost, variability in performance, and moderate specificity [14], and application of CT scan has some limitations, such as portability, intermittent use, and X-ray exposure [15].

Hence, there is a need for the development of reliable noninvasive and nonhazardous imaging techniques for the detection and characterization of breast cancer tumors.

Electrical impedance tomography (EIT) is a noninvasive technique to image the human breast using electrical impedance characteristics of the tissues [16]. In this imaging technique, an array of external electrodes is employed to extract and reconstruct an image of the inner part of a conductive object. The value of the electrical impedance in human tissues depends on the tissue's electrical storage potential. The electrical impedance is different in the normal tissues and pathologically changed tissues [17].

The variation of cellular water content, variation of extracellular fluids, packing density, and differences in the orientation of the cells are the main reasons for a significant increase in capacitance and conductance of malignant tumors, which results in lower impedance [18].

EIT is a rapid, compact, and inexpensive imaging solution [19–21]. The EIT technique can be employed with almost no age limitations. It can also be used on pregnant women because no ionizing radiation is emitted by the scanning device [16].

Several studies that have been performed previously focused on investigating the accuracy of EIT systems in the detection of breast cancer. These studies did not show comprehensive results in this field. However, to the best of the authors' knowledge, there are no comprehensive meta-analyses on this matter. On the other hand, from the clinical point of view, rare evidence is available for using this method for detecting breast cancer. So, there is still a gap between clinical use and research in this field.

Therefore, the aim of this manuscript is as follows: (1) a comprehensive investigation of the accuracy of the electrical impedance tomography technique for breast cancer diagnosis through searching evidence in the valid databases and (2) meta-analyses of the results.

## 2. Materials and Methods

### 2.1. Search Strategy and Information Source

A comprehensive literature search according to the Preferred Reporting Items for Systematic Reviews and Meta-Analyses (PRISMA) guidelines was performed. The search was performed in PubMed, Web of Science, EMBASE, Science Direct, ProQuest, Scopus, and Google Scholar search engine (without time limitation until January 2021) with no language restrictions.

The search syntax was developed based on Medical Subject Headings (MeSH) in PubMed. The keywords are ((Electrical Impedance Tomography) OR (EIT)) AND ((breast cancer) OR (breast neoplasm) OR (breast anomaly) OR (breast carcinoma) OR (breast lesions) OR (breast abnormality) OR (breast tumor) OR (breast palpable mass) OR (dense breast tissue) OR (Human Mammary Neoplasm) OR (Mammary cancer) OR (breast malignant neoplasm) OR (Breast malignant tumor)).

The reference lists of relevant primary studies, reviews, and key journals for additional studies were also searched.

### 2.2. Study Selection Process

This systematic review and meta-analysis were conducted based on the PRISMA statement. EndNote software manager was used to manage the references. After removing duplications, two authors independently reviewed references based on the title and abstract. We excluded narrative reviews, opinion pieces, letters, and any other publications lacking primary data. Any disagreements between the investigators regarding the inclusion of articles were discussed and resolved with a third reviewer.

### 2.3. Inclusion Criteria

Articles were included based on the following inclusion criteria:

1. Observational and interventional studies that assessed using the EIT to diagnose breast cancer
2. Published articles in any language with full English abstracts were considered
3. And explicitly calculated performance of the EII through performance metrics

### 2.4. Data Items

Two authors independently collected the data from articles and any conflicts were solved by the third author. The relevant data were extracted and collected in Excel form. Data items were author, year, study design, objectives, specificity, sensitivity, PPV, NPV, ACC, number of patients, age, TP, FP, FN, and TN.

### 2.5. Risk of Bias (Quality) Assessment

The quality of methodological of primary studies was assessed by the modified QUADAS-2 quality assessment tool which was developed by Whiting [22]. QUADAS is an 11-item generic tool that was developed specifically for implementation in diagnostic test accuracy reviews. QUADAS was developed using a formal consensus method informed by empirical evidence.

### 2.6. Effect Measures

In this study, the reference test was EIT and the gold standard was mammography. Specificity, sensitivity, ACC, and diagnostic odds ratio were the variables for the outcome measure.

We conducted Cook's distance analysis as a measure of the influence of a study on the model.

### 2.7. Statistical Analysis

The statistical analyses were performed by using STATA V.13 statistical software (Stata Corp. 2013. Stata Statistical Software: Release 13. College Station, TX: Stata Corp LP). The hierarchical/bivariate meta-analysis was performed with the “metandi” package for the ROC plot of sensitivity and specificity. Patient frequencies within extracted 2 × 2 data tables were implemented to generate the forest plot of the Accuracy index that was applied using the “metaprop” command, and double arcsine transformations were applied to stabilize the variance. Every study that did not have enough outcome variables was eliminated.

We evaluated the heterogeneity among the primary studies by the forest plots, *χ*2 test (assuming a significance at the a-level of 10%), and the I^2^ statistic for the Accuracy index.

## 3. Results

### 3.1. Study Characteristics

**Figure 1:**
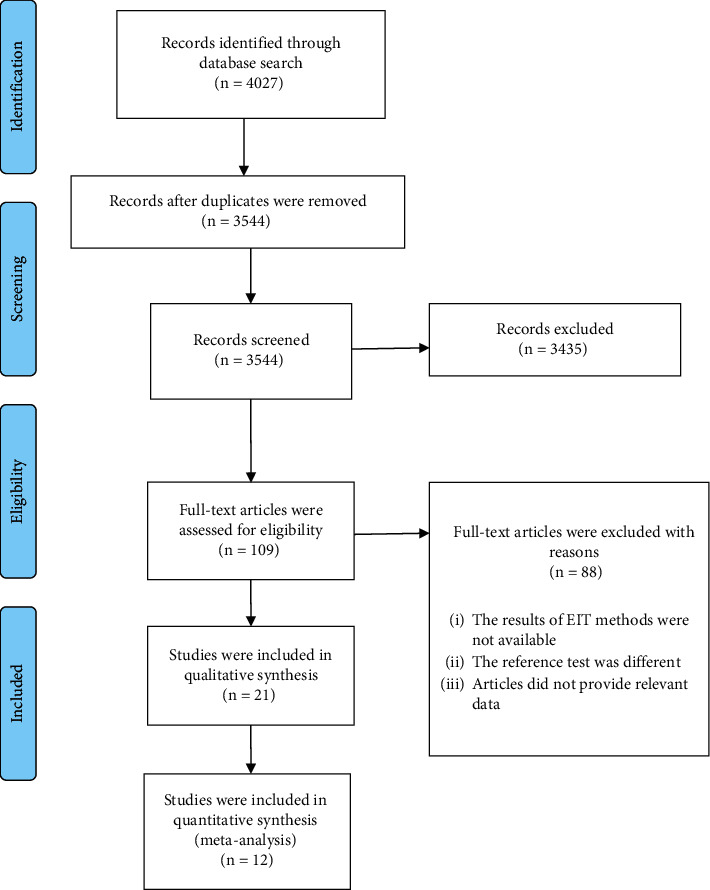
The flowchart illustrating the article search process and study inclusion.

A total of 4027 articles were initially retrieved from the electronic databases. After removing the duplicates, the remaining 3544 studies were screened. A total of 3435 studies were excluded. Among 109 remaining papers, 88 were excluded because their methods were not available or the reference test was different or it did not provide relevant data. Finally, 12 articles were selected for inclusion in this study.  Figure 1 illustrates the selection process of the articles based on the PRISMA flow diagram.

The 12 selected studies included data for 5487 patients with breast cancer. These studies were conducted between 1999 and 2017. Most of these studies were conducted in Germany, China, and the USA.

The main characteristics of each study and their sensitivity and specificity are summarized in Table 1.

### 3.2. Reporting Bias and Quality Assessment

According to QUADAS-2, the quality of the studies was evaluated. Each study was evaluated by two independent investigators. All disagreements were resolved by consensus. The quality of the included studies based on the QUADAS-2 tool showed a high risk of bias (Table 2). Seven articles had a high risk of bias, and 5 articles had a low risk of bias.

### 3.3. Meta-Analysis

**Figure 2:**
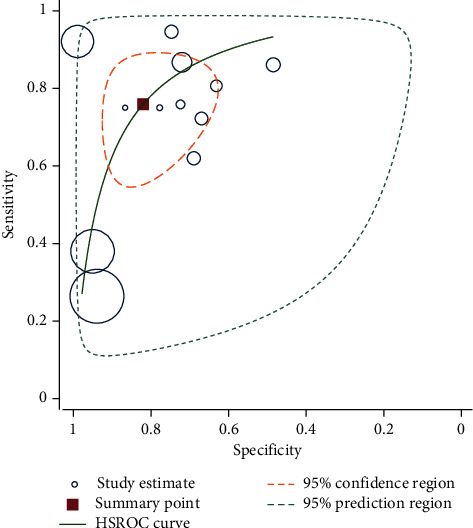
Summary ROC plot of sensitivity and specificity of EIT.

The summary ROC curve presents the summary estimates for EIT (Figure 2).

Table 3 illustrates the bivariate and the hierarchical summary receiver operating characteristic (HSROC) parameter estimates along with their standard errors and approximate 95% confidence intervals. Pooled sensitivity and specificity were 75.88 and 82.04 with the standard error of 6.16 and 5.14, respectively. The pooled diagnostic odds ratio (DOR) was 14.37 with a 6.14 standard deviation.

Figure 2 shows the summary ROC plot of sensitivity and specificity of EIT. This diagram is a graphical representation of a fitted model for simultaneous meta-analysis of Se and Sp on a ROC diagram. Each small circle of information is specific to a study, and the size of which is related to the weight assigned to it in the meta-analysis. The confidence interval and the prediction intervals are also marked with dashes. The pooled sensitivity and feature are also marked with a red dot. This graph uses the hierarchical summary receiver operating characteristic (HSROC) method to estimate results.

**Figure 3:**
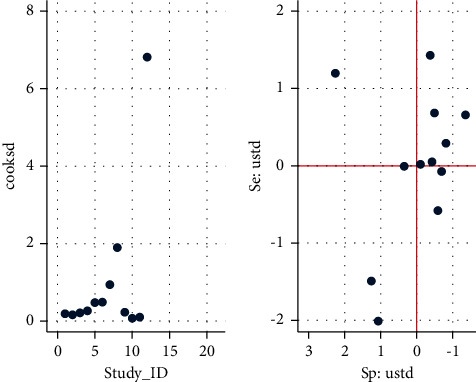
Left panel: Cook's distance. Right panel: standardized residuals (standardized predicted random effects).

Cook's distance analysis was conducted as a measure of the influence of a study on the model parameters [34]. The results indicate that two studies (ID numbers 8 and 12) had the most influence on the parameters (Figure 3).

Table 4 shows the results of the pooled effect of accuracy of 0.79 with 95% CI (0.73, 0.83) and *P* value = <0.01.

**Figure 4:**
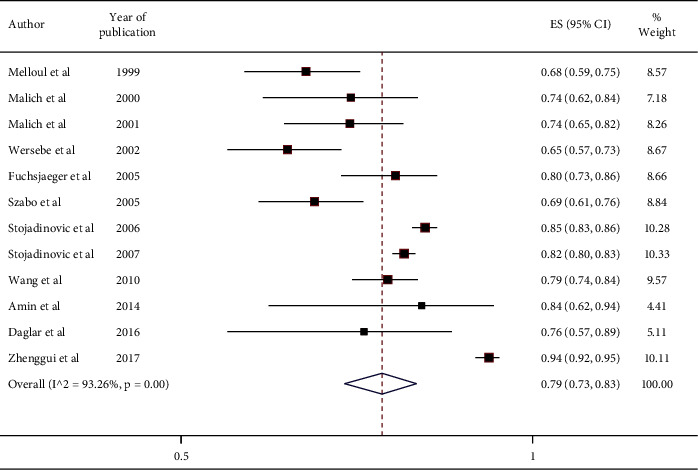
Forest plot of accuracy.

Significant heterogeneity was found between the studies (*I*^2^ = 93.26% and *P* value <0.01). The forest plot is shown in Figure 4. Due to the heterogeneity of the studies, the data were combined using a random-effect model. Different comparisons with different diagnostic methods may cause heterogeneity in our study.

**Figure 5:**
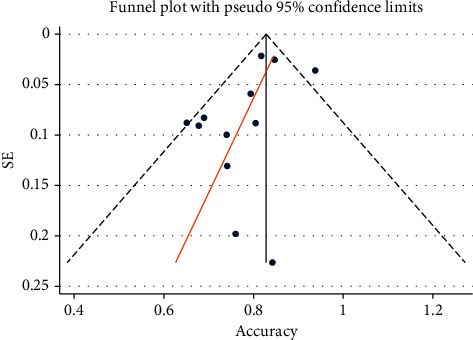
Funnel plot of accuracy.

Funnel plot was used to examine the publication bias, and the distribution of points in the two halves of the drawn triangle is discussed. Each point of the number is related to a study, which if these points are drawn relatively uniformly in all parts of the triangle, indicates the absence of publication bias. Finally, no significant publication bias was found through the funnel plot (*P* value = 0.108) (Figure 5).

## 4. Discussion

This study investigates the accuracy of EIT for detecting breast cancer. Most studies have reported several sensitivity and specificity values for the accuracy of anatomic EIT to diagnose breast cancer. The meta-analysis of 12 studies included in our research showed higher diagnostic accuracy for EIT (sensitivity and specificity of 75.88 ± 6.16 and 82.04 ± 5.14, respectively) and a DOR of 14.37 ± 6.14. The results of this meta-analysis revealed overall high sensitivity and specificity.

Based on our knowledge, our study is the first systematic review and meta-analysis to investigate the accuracy of the EIT for breast cancer detection. In this field, only one systematic review was performed by Zain et al. in 2015 and their review did not reach the meta-analysis. Their review showed that the range sensitivity of the EIT system to the human breast was between 17% and 94.6%. The range of specificity was between 49% and 97.1%. The accuracy (ACC) of EIT was between 69% and 80.5% [35].

Zhang in the meta-analysis of diagnostic accuracy of magnetic resonance imaging and mammography for breast cancer reported that MRI sensitivity was 0.92 (95% CI, [0.89, 0.94]) and specificity of 0.70 (95% CI, [0.66, 0.73]) [36]. While Medeiros reported MRI pooled sensitivity of 90% (95% CI, 88–92%) and specificity of 75% (95% CI, 70–79%), respectively [37], and Xiang 0.97 (95% CI, 0.95–0.98) and 0.52 (95% CI, 0.46–0.58) as well [38]. So, we can say EIT sensitivity could not be compared with the sensitivity of MRI, but in terms of specificity, it can be considered as a new method that probably can get more attention. However, the stage of tumor and grade may affect these results.

Although MRI is an accepted tool in detecting tumors, breast MRI is expensive and can only diagnose breast lesions in certain clinical situations [39]. As such, alternative devices could be favorable. EIT is a radiation-free and noninvasive method that can be employed as an inexpensive, portable, and radiation-free technique for imaging [20, 21]. Hence, it is a great candidate for bedside diagnosis and intraoperative imaging applications [40]. However, EIT was not acceptable for clinical diagnosis until now, and more research is needed for its development.

Several studies were conducted using meta-analyses for mammography accuracy stand-alone or in comparison with other imaging techniques. The range of pooled sensitivity of 75–97% and the specificity of 66–96% with 95% CI [36, 38, 41] was reported. The sensitivity of 75% and specificity of 82% of our study showed that EIT can be used as a useful method alongside mammography.

In comparison with ultrasound or mammography, EIT is less effective due to its low specificity [27]. It appears that age is an important determinative factor in this regard. As seen in studies [29, 30], EIT may have more efficiency for people who are 30–45 years old. Breast cancers in young women are usually diagnosed at a more advanced stage, requiring more serious and expensive treatments with a reduction in the survivorship rate and the quality of life [42]. This late diagnosis is due to the lack of mammography evaluation for people under 40 years old. In addition, small and nonpalpable probably benign lesions remain unnoticed with either clinical, self-breast examination, or screening surveillance until they have grown sufficiently large enough that the woman herself detects a palpable mass [42, 43]. Therefore, for people under 40, the use of low-cost modalities with acceptable sensitivity and specificity at the initial stages of the disease is required to reduce the rate of unnecessary biopsies [42].

Screening surveillance in the probably benign lesions is more cost-effective than surgical intervention and prevents unnecessary biopsies [44]. However, despite all efforts for follow-ups, approximately 30 to 47% of participants did not complete the screening surveillance [45, 46]. EIT has demonstrated good results in detecting nonpalpable breast cancer as a safe diagnostic tool in younger women [42].

Subgroup analysis can provide more specific information for diagnosis in special groups. In this study, we did not perform subgroup analysis because of limited initial information in selected studies. Respectively, discrimination between patients of different ages was not possible, for example, in patients under 40, the sensitivity of mammography to detect cancer is decreased [47].

Different quality of articles may affect the result of meta-analysis. However, it showed that the use of EIT can be useful because of its sensitivity in detecting cancer in cases that were not detected by mammography and ultrasound [48].

### 4.1. Limitations of the Study

1. Unfortunately, it is not possible to analyze the histopathology of patients to determine the efficiency of EIT in detecting the type of different mass in young women
2. Different comparisons with different diagnostic methods may cause heterogeneity in our study
3. Insufficient information and unclear mean age in different groups in articles

## 5. Conclusion

This paper demonstrated an acceptable accuracy of EIT for breast cancer detection. However, it is concluded that ETL is more considered in the research and clinical application was not very clear yet. This study showed that EIT can be used as a useful method alongside mammography. EIT sensitivity could not be compared with MRI, but in terms of specificity, it can be considered as a new method that probably can get more attention. Furthermore, large-scale studies will be needed to support the evidence.

## Figures and Tables

**Table 1:** Summary of studies included in the meta-analysis.

ID	Author	Year	Sample size	Age of participants	Country	Sensitivity %	Specificity %
1	Melloul et al. [23]	1999	121	42 to 70	Israel	72.2	67
2	Malich et al. [24]	2000	58	—	Germany	75.9	72.4
3	Malich et al. [25]	2001	100	—	Germany	81	63
4	Wersebe et al. [26]	2002	129	Mean = 55	Germany	62	69
5	Fuchsjaeger et al. [27]	2005	128	21 to 89	Austria	94.6	74.7
6	Szabo et al. [28]	2005	145	33 to 92	Sweden	86	49
7	Stojadinovic et al. [29]	2006	1550	30 to 45	USA	38	95.1
8	Stojadinovic et al. [30]	2007	2155	30 to 45	USA	26.4	94.7
9	Wang et al. [31]	2010	286	25 to 45	China	86.7	72.9
10	Amin et al. [7]	2014	19	17 to 55	Bangladesh	75	87
11	Daglar et al. [32]	2016	25	18 to 85	Turkey	75	77.78
12	Zhenggui et al. [33]	2017	771	18 to 83	China	92.1	98.9

**Table 2:** QUADAS for the included studies.

ID	Author	QUADAS questions						
		Risk of bias				Applicability concern		
		Patientselection	Referencestandard	Index test	Flow andtiming	Patientselection	Index test	Referencestandard
1	Melloul et al. [23]	Yes	Yes	Yes	Yes	No	Yes	Yes
2	Malich et al. [24]	Yes	No	Yes	Yes	?	Yes	Yes
3	Malich et al. [25]	Yes	No	Yes	Yes	Yes	No	Yes
4	Wersebe et al. [26]	Yes	No	Yes	Yes	Yes	No	Yes
5	Fuchsjaeger et al. [27]	Yes	Yes	Yes	Yes	?	Yes	Yes
6	Szabo et al. [28]	Yes	Yes	Yes	Yes	Yes	Yes	Yes
7	Stojadinovic et al. [29]	Yes	No	Yes	Yes	Yes	No	Yes
8	Stojadinovic et al. [30]	Yes	No	Yes	Yes	Yes	No	Yes
9	Wang et al. [31]	Yes	Yes	Yes	Yes	Yes	Yes	Yes
10	Amin et al. [7]	Yes	Yes	Yes	Yes	?	Yes	Yes
11	Daglar et al. [32]	Yes	No	Yes	Yes	Yes	Yes	Yes
12	Zhenggui et al. [33]	Yes	No	Yes	Yes	Yes	Yes	Yes

**Table 3:** Meta-analysis of diagnostic accuracy.

	Parameter estimation	SE	95% CI	
Sensitivity	75.88	6.16	61.92	85.89
Specificity	82.04	5.14	69.72	90.06
DOR^*∗*^	14.37	6.14	6.22	33.20

^*∗*^Diagnostic odds ratio.

**Table 4:** Meta-analysis of accuracy.

Study	Accuracy	95% CI		Weight (%)
Melloul et al. [23]	0.68	0.59	0.75	8.57
Malich et al. [24]	0.74	0.62	0.84	7.18
Malich et al. [25]	0.74	0.65	0.82	8.26
Wersebe et al. [26]	0.65	0.57	0.73	8.67
Fuchsjaeger et al. [27]	0.80	0.73	0.86	8.66
Szabo et al. [28]	0.69	0.61	0.76	8.84
Stojadinovic et al. [29]	0.85	0.83	0.86	10.28
Stojadinovic et al. [30]	0.82	0.80	0.83	10.33
Wang et al. [31]	0.79	0.74	0.84	9.57
Amin et al. [7]	0.84	0.62	0.94	4.41
Daglar et al. [32]	0.76	0.57	0.89	5.11
Zhenggui et al. [33]	0.94	0.92	0.95	10.11
Pooled effect	0.79	0.73	0.83	100

## Data Availability

The data used to support the findings of this study are available from the corresponding author upon request.
